# Finger and forehead photoplethysmography-derived pulse-pressure variation and the benefits of baseline correction

**DOI:** 10.1007/s10877-018-0140-5

**Published:** 2018-04-11

**Authors:** Shaoxiong Sun, Wouter H. Peeters, Rick Bezemer, Xi Long, Igor Paulussen, Ronald M. Aarts, Gerrit J. Noordergraaf

**Affiliations:** 1 Department of Electrical Engineering, Eindhoven University of Technology, Eindhoven, The Netherlands; 2 Philips Research, Eindhoven, The Netherlands; 3 Elisabeth-Tweesteden Hospital, Tilburg, The Netherlands

**Keywords:** Fluid responsiveness, Volume status, Pulse pressure variation, Photoplethysmography, Site dependency, Major surgery

## Abstract

To non-invasively predict fluid responsiveness, respiration-induced pulse amplitude variation (PAV) in the photoplethysmographic (PPG) signal has been proposed as an alternative to pulse pressure variation (PPV) in the arterial blood pressure (ABP) signal. However, it is still unclear how the performance of the PPG-derived PAV is site-dependent during surgery. The aim of this study is to compare finger- and forehead-PPG derived PAV in their ability to approach the value and trend of ABP-derived PPV. Furthermore, this study investigates four potential confounding factors, (1) baseline variation, (2) PPV, (3) ratio of respiration and heart rate, and (4) perfusion index, which might affect the agreement between PPV and PAV. In this work, ABP, finger PPG, and forehead PPG were continuously recorded in 29 patients undergoing major surgery in the operating room. A total of 91.2 h data were used for analysis, from which PAV and PPV were calculated and compared. We analyzed the impact of the four factors using a multiple linear regression (MLR) analysis. The results show that compared with the ABP-derived PPV, finger-derived PAV had an agreement of 3.2 ± 5.1%, whereas forehead-PAV had an agreement of 12.0 ± 9.1%. From the MLR analysis, we found that baseline variation was a factor significantly affecting the agreement between PPV and PAV. After correcting for respiration-induced baseline variation, the agreements for finger- and forehead-derived PAV were improved to reach an agreement of − 1.2 ± 3.8% and 3.3 ± 4.8%, respectively. To conclude, finger-derived PAV showed better agreement with ABP-derived PPV compared to forehead-derived PAV. Baseline variation was a factor that significantly affected the agreement between PPV and PAV. By correcting for the baseline variation, improved agreements were obtained for both the finger and forehead, and the difference between these two agreements was diminished. The tracking abilities for both finger- and forehead-derived PAV still warrant improvement for wide use in clinical practice. Overall, our results show that baseline-corrected finger- and forehead-derived PAV may provide a non-invasive alternative for PPV.

## Introduction

Several studies have demonstrated that intraoperative hemodynamic optimization using goal-directed volume expansion reduces postoperative morbidity and hospital stay in high-risk surgery patients [1–3]. To guide volume management, static preload indices, such as central venous pressure, were proposed, but their values have been questioned [4, 5]. Dynamic indices depicting preload dependence, on the other hand, have shown superior performance [6, 7]. Among these dynamic indicators, pulse pressure variation (PPV) has been shown to achieve the highest sensitivity and specificity [8].

Measuring PPV usually requires arterial catheterization and therefore has a risk of causing medical complications. This has driven the emergence of a noninvasive alternative derived from photoplethysmography (PPG) [9]. In line with PPV, the pulse amplitude variation (PAV) of the PPG signal serves the same function. Although some studies show good correlations between PPV and PAV [10–12], the others report poor results especially those investigating their relationship over a long period of time [13–15].

Several explanations have been provided to account for the discrepancies in the findings, such as the measurement site, signal processing algorithm embedded in the monitor, or oscillation of the sympathetic nerve activity, and administration of vasopressors [16]. One suggestion was that PPG sensors placed in the cephalic region might improve the relationship between PPV and PAV as this area is less sensitive to changes in vasomotor tone and allow a stronger expression of ventilation effects [17–19].

To the best of our knowledge, only one study has addressed site dependency in the relationship between PPV and PAV, where measurements were performed before and after volume expansion in well-controlled situations prior to surgery [20]. The relationship between PPV and PAV at multiple sites during ongoing surgery, where patients undergo hemodynamic changes, remains unknown. Thus, our aim was to investigate and compare the ability of PAV, measured at the finger and on the forehead, to approach the value and trend of PPV in mechanically-ventilated patients undergoing major surgery. In addition, we studied four potential confounding factors, (1) baseline variation, (2) PPV, (3) ratio of respiration and heart rate, and (4) perfusion index (PI), which might affect the agreement between PPV and PAV in order to gain insights into the underlying mechanisms that limit the agreement between PPV and PAV.

## Materials and methods

### Patients

The study was reviewed and approved by the regional medical ethics committee (METC Brabant, The Netherlands, NL48421.028.14-P1409). With written informed consent, a heterogeneous group of 29 patients scheduled for major surgery was enrolled. Characteristics of patients is shown in Table 1.

**Table 1:** Patient characteristics (n = 29)

Age [year]	70.0 ± 8.9
Gender (male/female)	23/6
BMI [kg/m^2^]	27.8 ± 9.7
Height [cm]	172.3 ± 13.7
Length of operation [hours]	4.4 ± 1.4
Surgical procedures	
Urology	
Bricker deviation	14
Radical prostatectomy	3
Cystectomy	1
Pyeloplasty	1
Vascular surgery	
FEM-Fem bypass or crossover	4
EVAR removal and replacement	3
PTA femoral artery	1
Recanalization iliac artery	1
Carotid endarterectomy	1

### Protocol

We used the same dataset as in the previous work [21]. Anesthesia was induced by propofol (2 mg/kg), sufentanil (0.5 mg/kg), and rocuronium (0.6 mg/kg), and maintained by means of continuous infusion of sufentanil and propofol. The depth of anesthesia was assessed using bispectral index (an EEG-based parameter for assessing depth of hypnosis) with a target of 40–55. The patients were ventilated in a volume-controlled, pressure-limited mode with tidal volume of 6–10 ml/kg at a frequency of 10–14/min, and adjusted to maintain normocapnea. The positive end-expiratory pressure was set at 6 cm H_2_O and adjusted as needed. Fluid management was at the discretion of the physician. The hemodynamic management was controlled by general instructions to the anesthesiologist: strive to maintain reference blood pressure within the range of good clinical practice 9 ± 15% of reference. Based on the available data, the anesthesiologist could typically (a) give bolus volume: crystalloid, (b) give bolus phenyl-ephedrine (100 mg) or ephedrine (5 or 7.5 mg), and/or (c) start phenyl-ephedrine in continuous infusion. Blood products were given as needed to match the 4–5–6 rule in which physicians are guided along other parameters then the hemoglobin before the decision is made to transfuse a patient [22]. Combinations were also possible. During surgery, three signals were collected: invasive ABP signals (Philips Heartstart MRx monitor) by a radial arterial catheter, finger PPG signals obtained at the right index finger (Philips M1191B), and forehead PPG signals (Covidien MaxFast). The PPG probes were attached to the patient in the holding. Probes were visually controlled according the instructions for use and checked after movement to the OR, and checked during the procedures. No extra attachments or shielding was performed. The conditions of all patients can be found in Table 2 (six patients were removed as described in the follow section).

**Table 2:** Patient conditions (n = 23)

Patient no	Body temperature [min max]	Blood loss (ml)	Infusion (ml)					Hemoglobin (mmol/l) [min max]	Mean blood pressure [mean ± SD]
			NaCl 0.9%	Volu^a^	Ery^b^	RL^c^	FFP^d^		
1	[35.1 36.6]	1700	4993	1436	–	–	–	[5.6 6.4]	[66.6 ± 12.2]
2	[35.3 36.4]	2400	2997	730	1326	–	578	[5.4 7.4]	[76.7 ± 9.5]
3	[35.3 36.7]	800	1840	924	–	2044	–	[6.3 6.9]	[88.5 ± 11.2]
4	[35.3 36.7]	5000	4485	962	1026	3971	568	[4.5 6.2]	[73.3 ± 17.9]
5	[35.6 36.6]	2400	3482	942	526	2482	–	[6.6 7.5]	[71.8 ± 10.4]
6	[34.1 36.4]	1400	4000	944	783	3488	–	[5.0 7.3]	[65.3 ± 13.1]
7	[34.3 36.2]	4600	6491	1378	1347	1990	–	[4.9 6.5]	[69.7 ± 9.8]
8	[35.0 36.8]	1000	2997	988	534	1995	–	[5.5 6.5]	[86.5 ± 14.7]
9	[34.2 35.7]	2200	2998	976	512	2493	–	[5.5 8.6]	[67.9 ± 11.0]
10	[35.8 36.5]	3000	2535	923	794	4996	–	[5.9 7.3]	[62.7 ± 15.6]
11	[34.6 35.5]	1000	2791	469	–	1493	–	[7.0 8.0]	[65.6 ± 7.2]
12	[34.4 35.5]	2400	5991	1316	265	1998	–	[5.5 7.5]	[72.3 ± 11.9]
13	[34.4 35.6]	3500	2998	925	787	1982	–	[4.8 6.3]	[62.7 ± 8.0]
14	[35.2 36.6]	2900	3995	–	528	2921	–	[5.0 6.9]	[78.0 ± 16.4]
15	[35.5 35.8]	600	3495	485	–	–	–	[7.4 7.4]	[57.4 ± 6.6]
16	[36.5 36.9]	600	1786	–	–	–	–	[7.9 8.4]	[72.4 ± 7.5]
17	[35.0 36.0]	1100	2496	977	–	–	–	[5.7 8.0]	[66.0 ± 9.9]
18	[35.0 36.1]	400	3992	483	268	–	–	[4.6 6.1]	[87.3 ± 12.8]
19	[34.5 35.3]	150	3981	492	–	–	–	[5.8 6.9]	[66.3 ± 6.6]
20	[34.1 36.3]	700	986	974	–	3046	–	[5.4 6.9]	[67.1 ± 7.6]
21	[34.8 36.0]	1300	5492	477	–	1498	–	[6.0 7.8]	[68.7 ± 8.9]
22	[34.6 35.5]	1100	5141	954	–	496	–	[5.0 6.4]	[65.1 ± 9.1]
23	[34.9 36.4]	2400	*	*	*	*	*	[4.3 5.2]	[61.9 ± 10.0]

*Data not recorded

^a^Voluven

^b^Erythrocyte

^c^Ringers lactate

^d^Fresh frozen plasma

### Data analysis

**Fig. 1:**
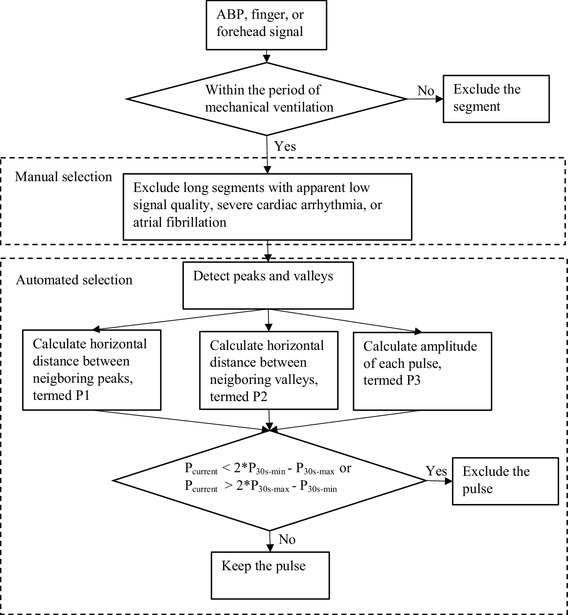
The diagram of signal selection process

Figure 1 gives an illustration of the signal selection process. Signal analysis was confined to the period of mechanical ventilation. Signal segments with low signal quality, severe cardiac arrhythmia, or atrial fibrillation were excluded by manual selection and a dedicated program. In manual selection, relatively long problematic segments were removed. This was followed by the operation of the dedicated program. This program, after identifying peaks and valleys for each pulse, computed three parameters: the horizontal distance between neighboring peaks, the horizontal distance between neighboring valleys and the amplitude of each pulse. For each parameter, if the difference between the present value and the extrema (maximum or minimum) in the 30 s history window prior to that pulse was larger than the discrepancy between these maximum and minimum values, this pulse was excluded. As a result, data from 6 patients were removed entirely and from the remaining 23 patients, 91.2 h of data were found eligible for further analysis of PPV (9.8% was excluded due to poor signal quality or cardiac arrhythmia). Note that an eligible segment require all three signals be of acceptable signal quality simultaneously.

The length of a ventilation cycle was derived using the respiration rate readily available from the ventilator. As signals were, in most cases, short-term stationary (the frequency components remain unchanged), the exact start and end of one ventilation cycle were not necessary as long as the length was correctly determined. In this work, we used the starting time of mechanical ventilation as the onset of the first ventilation cycle.

PPV was calculated as described by Michard et al. [23]:

$${\text{PPV }} = {{\left( {{\text{PP}}_{{\max }} - {\text{ PP}}_{{\min }} } \right)} \mathord{\left/ {\vphantom {{\left( {{\text{PP}}_{{\max }} - {\text{ PP}}_{{\min }} } \right)} {\left[ {\left( {{\text{ PP}}_{{\max }} + {\text{ PP}}_{{\min }} } \right)/2} \right]}}} \right. \kern-\nulldelimiterspace} {\left[ {\left( {{\text{ PP}}_{{\max }} + {\text{ PP}}_{{\min }} } \right)/2} \right]}},$$

where PP stands for pulse pressure and the subscripts max and min indicate the corresponding maximal and minimal values during each ventilation cycle, respectively.

Similarly, PAV was calculated according to the paper by Cannesson et al. [24]:

$${\text{PAV }} = {{\left( {{\text{PA}}_{{{\text{max}}}} - {\text{ PA}}_{{{\text{min}}}} } \right)} \mathord{\left/ {\vphantom {{\left( {{\text{PA}}_{{{\text{max}}}} - {\text{ PA}}_{{{\text{min}}}} } \right)} {\left[ {\left( {{\text{ PA}}_{{{\text{max}}}} + {\text{ PA}}_{{{\text{min}}}} } \right)/2} \right]}}} \right. \kern-\nulldelimiterspace} {\left[ {\left( {{\text{ PA}}_{{{\text{max}}}} + {\text{ PA}}_{{{\text{min}}}} } \right)/2} \right]}},$$

where PA stands for PPG waveform amplitude. The derived PPV and PAV values were smoothed using a 5-point median filter. To suppress time dependency in the statistical analysis, we down-sampled PPV and PAV values by a factor of 20. This means that PPV and PAV data points were generated every 20 ventilation cycles.

In addition to site dependency, four potential confounding factors were studied, which could affect the relationship between PPV and PAV: (1) baseline variation, (2) PPV, (3) ratio of respiration and heart rate, and (4) perfusion index (PI).

The first factor studied was baseline variation. The Baseline in the PPG signal, independently of PA, is also modulated by the ventilation [17]. When the baseline modulation is so strong that the pulse peak and pulse valley are influenced differently, the derived pulse amplitude is inaccurate, compromising the calculation of PPV or PAV. Thus, it is important to study the influence of the baseline modulation on the agreement between PPV and PAV. In the literature, the baseline modulation has been extracted as ratio between the power at the ventilation frequency and the power at the cardiac frequency [25]. However, physiological signals are often long-term non-stationary during surgery, causing a violation of assumptions on the stationarity of the Fourier Transform used for frequency analysis. To address this, we introduced a new index for baseline variation, termed BV, which is derived in the time domain. This index was defined as the variations in the diastolic value of each pulse over the mean pulse amplitude for each ventilation cycle. We computed the mean of BV for each patient.

In addition to BV, we also studied the influence of the PPV, the ratio of the respiration rate and the heart rate, and perfusion index on the agreement between PPV and PAV, as these were found or assumed to have an impact in previous work [13, 14, 20]. In this work, PI was calculated as ratio between the pulsatile (AC) component and the slowly-changing (DC) component. The three means of these features were computed for each patient, respectively.

**Fig. 2:**
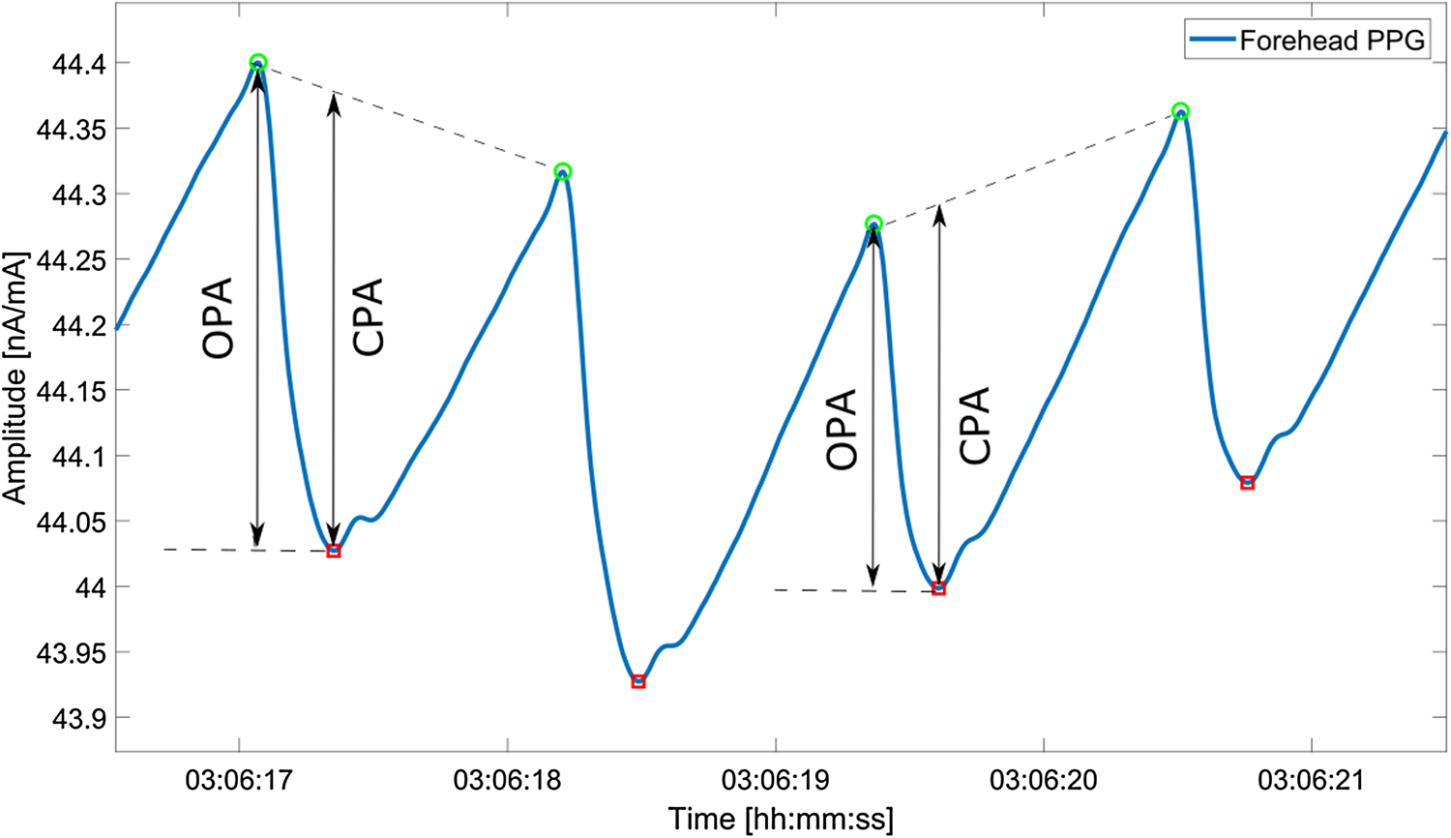
An example of baseline correction. The segment length is approximately one ventilation cycle (5 s). The original extrema are denoted by OPA (original pulse amplitude), while the corrected extrema are denoted by CPA (corrected pulse amplitude)

In an attempt to correct for the effect of BV on the PAV, we also computed a baseline-corrected form of the PAV. As shown in Fig. 2, before baseline correction, two original pulse amplitudes (OPA) were used for calculating PAV. After baseline correction, two corrected pulse amplitudes (CPA) were used for calculating PAV. The baseline-corrected PAV was computed in the same way as the uncorrected PAV, except that the baseline modulation in the PPG signal was diminished.

### Statistics

Bland–Altman analysis was performed to assess the agreement between PPV and PAV. This analysis was first done by aggregating all data points of all patients, and also for each patient individually. To evaluate how PAV tracks the changes in PPV, we used the four-quadrant plot method [26] on the aggregated data from all patients.

In the four-quadrant plot, each data point represents simultaneous changes in the two variables PPV and PAV, which are derived by their differentiation, respectively. Points falling into either the first or third quadrant indicate that the two variables change in the same direction, e.g. either increase or decrease concurrently. A concordance rate is defined as the ratio of the number of these points and the total number of points. A rate higher than 90% is regarded as a reliable trending ability provided that points around the center of the plot, often caused by noise, are excluded from the analysis. In this study, the central exclusion zone was set to be 2%.

In order to investigate the influence of each of the four factors, we applied multiple linear regression (MLR) analysis to show whether they had significant linear dependency on the agreements between PPV and PAV. We assigned the mean and SD of difference to dependent variables for each patient. If the regression coefficient associated with one variable is significantly non-zero (p < 0.05), this variable is regarded to have a significant association with the dependent variable.

## Results

**Fig. 3:**
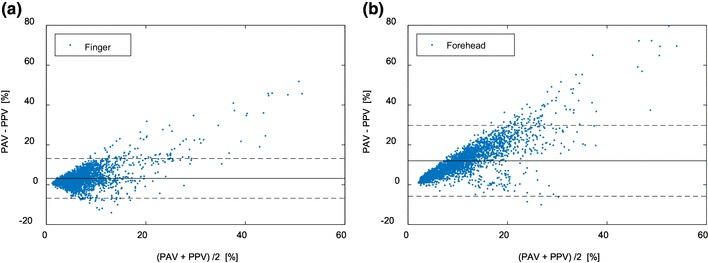
Bland–Altman plot of PPG-derived PAV versus ABP-derived PPV for the data points of all patients aggregated. **a** Finger-derived PAV versus PPV. **b** Forehead-derived PAV versus PPV. The solid line corresponds to the bias (mean difference) and the dotted lines correspond to the limits of agreement (1.96 × SD of difference)

Figure 3 shows the Bland–Altman plots of finger- and forehead-derived PAV versus PPV for the aggregated data from all patients. While the difference between PPV and PAV increases with their averages for the forehead-derived PAV, this effect is less pronounced for the finger-derived PAV. Table 3 also shows that the finger-derived PAV agreed better with PPV than forehead-derived PAV, as can be seen in both the mean and SD of the difference. Concordance rate differed only marginally between finger and forehead PPG.

**Table 3:** Comparison between PPV and PPG-derived PAV

	Finger	Forehead
Mean ± SD^a^ of difference (agreement)	3.2 ± 5.1%	12.0 ± 9.1%
Correlation coefficients	0.70	0.60
Concordance rate	84%	83%

^a^*SD* standard deviation

Table 4 presents individual agreements for both finger and forehead PPG for all 23 patients. It can be seen that PAV was consistently higher than PPV for both sites and for almost all patients. Tables 5 and 6 show the effect of the four potential confounding factors on the mean and SD of difference between PAV and PPV. It can be seen that BV most significantly influenced the agreement for both finger and forehead PPG.

**Table 4:** Bland–Altman analysis comparing PAV and PPV for each patient individually

Patient	Mean ± SD^a^ of difference (agreement)		Patient	Mean ± SD^a^ of difference (agreement)	
	Finger (%)	Forehead (%)		Finger (%)	Forehead (%)
1	6.7 ± 4.4	17.7 ± 8.6	13	4.0 ± 5.2	11.3 ± 7.5
2	3.6 ± 4.2	14.6 ± 8.1	14	4.0 ± 8.7	7.5 ± 9.7
3	2.8 ± 5.8	7.6 ± 8.1	15	2.7 ± 5.2	11.3 ± 6.9
4	7.1 ± 7.2	9.3 ± 6.6	16	2.4 ± 3.5	11.8 ± 5.1
5	− 1.7 ± 3.3	12.4 ± 6.7	17	15.8 ± 11.9	5.1 ± 1.0
6	2.1 ± 3.2	5.0 ± 3.6	18	7.1 ± 6.1	9.2 ± 5.3
7	2.3 ± 5.1	17.0 ± 12.5	19	2.2 ± 2.9	9.3 ± 4.1
8	4.5 ± 6.6	14.7 ± 13.3	20	2.5 ± 3.5	9.4 ± 9.6
9	1.4 ± 3.8	5.6 ± 8.3	21	6.5 ± 7.7	3.6 ± 4.2
10	1.5 ± 9.0	22.6 ± 12.4	22	3.1 ± 4.9	16.0 ± 12.0
11	0.5 ± 4.8	17.0 ± 11.5	23	− 1.4 ± 4.5	21.4 ± 5.1
12	3.2 ± 4.3	17.2 ± 10.8			

^a^*SD* standard deviation

**Table 5:** Multiple linear regression coefficients of the mean difference between PPV and PAV on four potentially confounding factors

	Finger		Forehead	
	Coefficient	P value	Coefficient	P value
BV^a^	1.85	0.05	3.59	0.005*
PPV	0.22	0.77	0.24	0.83
HR RR ratio^b^	− 0.11	0.89	− 2.21	0.02*
PI^c^	− 0.68	0.35	− 1.44	0.13

*Statistically significant P < 0.05

^a^*BV* baseline variation

^b^*HR RR ratio* the ratio between heart rate and respiration rate

^c^*PI* perfusion index

**Table 6:** Multiple linear regression coefficients of the SD of the difference between PPV and PAV on four potential confounding factors

	Finger		Forehead	
	Coefficient	P value	Coefficient	P value
BV^a^	1.09	0.04*	1.61	0.05
PPV	0.97	0.03*	0.12	0.88
HR RR ratio^b^	− 0.72	0.12	− 0.90	0.17
PI^c^	− 0.38	0.36	− 0.63	0.33

*Statistically significant p < 0.05

^a^*BV* baseline variation

^b^*HR RR ratio* the ratio between heart rate and respiration rate

^c^*PI* perfusion index

The individual statistics is reported as median (interquartile 25th–75th percentile). The median BV was 0.04 (0.03–0.04), 0.13 (0.09–0.19), and 0.27 (0.20–0.31) for ABP, finger-derived PPG, and forehead-derived PPG, respectively (all significantly different from each other). This indicates that the baseline modulation in the PPG signal is significantly stronger than that in the ABP signal. This is more pronounced in the forehead PPG signal.

**Fig. 4:**
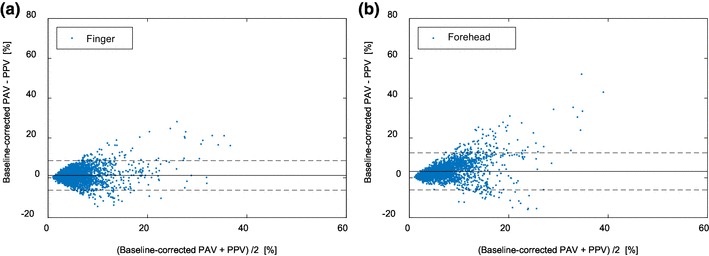
Bland–Altman plot of PPG-derived PAV versus ABP-derived PPV. **a** Finger-derived PAV versus PPV. **b** Forehead-derived PAV versus PPV. The solid line corresponds to the bias (mean difference) and the dotted lines correspond to the limits of agreement (1.96 × SD of the difference)

**Fig. 5:**
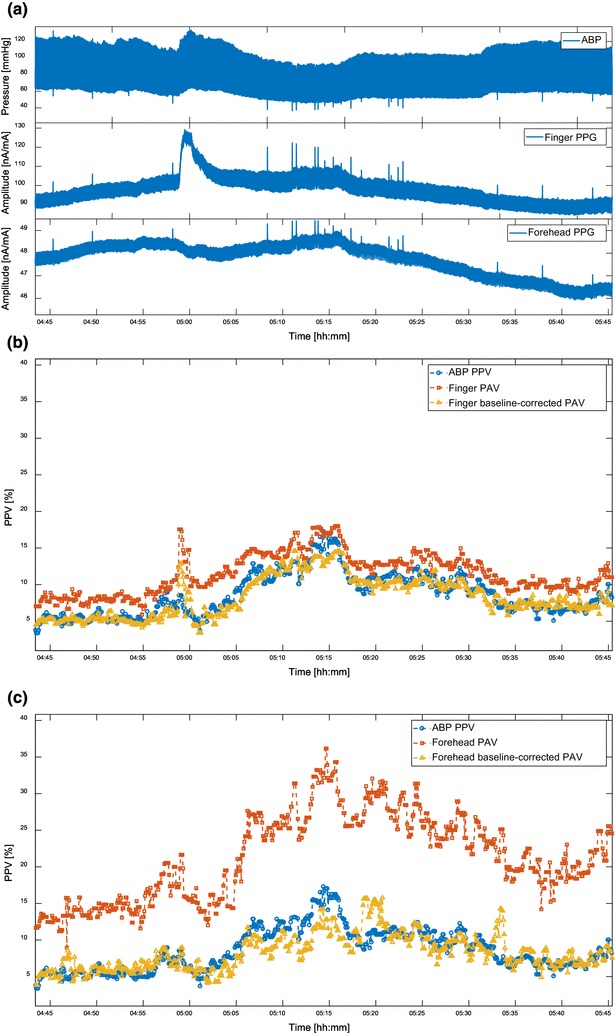
An example of how baseline correction helps improve the agreement between PAV and PPV. **a** ABP, finger PPG, and forehead PPG signals. **b** ABP-derived PPV, finger-derived PAV, baseline-corrected finger-derived PAV. **c** ABP-derived PPV, forehead-derived PAV, baseline-corrected forehead-derived PAV

**Fig. 6:**
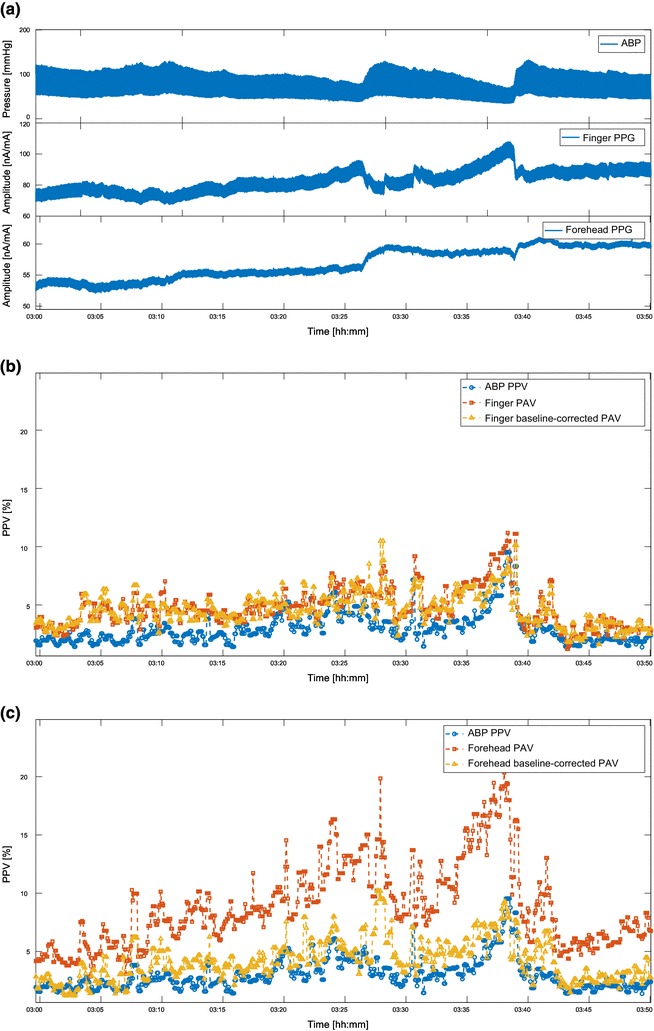
An example of the behavior of PAV in comparison to PPV in the episodes with fast hemodynamic changes. **a** ABP, finger PPG, and forehead PPG signals. **b** ABP-derived PPV, finger-derived PAV, baseline-corrected finger-derived PAV. **c** ABP-derived PPV, forehead-derived PAV, baseline-corrected forehead-derived PAV

Figure 4 shows the Bland–Altman plot of baseline-corrected finger- and forehead-derived PAV versus PPV. Compared to Fig. 3, the BV correction reduced the difference between PAV and PPV over the entire PPV range measured. Table 7 shows the improvements in the agreement between PPV and PAV at both sites. The agreement between forehead-PAV and PPV was improved from 12.0 ± 9.1% to 3.3 ± 4.8%, and the agreement between finger-PAV and PPV improved from 3.2 ± 5.1 to 1.2 ± 3.8%. It should also be noted that other performance parameters changed only marginally. Figure 5 gives an example of how BV correction helped improve the agreement. For the sake of simplicity and clarity, PPV and PAV values were further smoothed using a 10-point moving average filter. It can be seen that after the baseline correction, the agreement was improved for both finger- and forehead-PAV. Figure 6 illustrates the behavior of PAV in comparison to PPV in the episodes with fast hemodynamic changes. It is shown that baseline-corrected PAV was able to approach the value and trend of PPV in this scenario.

**Table 7:** Comparison between PPV and PPG-derived PAV with correction for baseline variation

	Finger	Forehead
Mean ± SD^a^ of difference (agreement)	1.2 ± 3.8%	3.3 ± 4.8%
Correlation coefficients	0.64	0.61
Concordance rate	81%	82%

^a^*SD* standard deviation

## Discussion

In this study, we investigated the ability of PPG-derived PAV to approach the value and the trend of PPV for both finger and forehead PPG during surgery. We found that initially the PAV derived from finger PPG showed a much better agreement with PPV in comparison to PAV derived from forehead PPG. By correcting for BV, we improved the agreement between PPV and PAV for both PPG-measurement sites. The difference in the agreement was greatly diminished. Moreover, we did not see significant discrepancy in trending ability for finger- and forehead-derived PPG with and without BV correction.

The presence of baseline modulations in the PPG signal has been described by Shelley et al. [17, 25]. Moreover, its impact on PAV was discussed recently in a simulation by Høiseth et al. [27]. In the present work, we demonstrated, by computing and comparing BV, that the baseline modulation in the ABP signal was much weaker than that in the PPG signals. Furthermore, we showed that the baseline modulation manifested itself more strongly in the forehead than in the finger, which is in line with the finding by Shelley et al. [17]. In fact, the increase in BV from the ABP signal to the finger and forehead PPG signals might be caused by the presence of venous component in the PPG signals [17, 28]. Finally, we showed that BV was a factor confounding the agreement between PPV and PAV. By correcting for BV, we achieved better agreements for both sites.

In addition to BV, we also investigated three other potential confounding factors (PI, HR-RR ratio, and PPV mean). Previously, finger PI has been shown to affect the agreement between PAV and PPV [29]. In their work, to correct for this, finger-derived PAV values were reduced artificially when the finger was poorly perfused (PI < 3%). We did not observe that the agreement between PAV and PPV was significantly affected by the PI. We speculate that this might be attributed to a lack of poorly-perfused patients in this study, as only 3 out of 23 patients had a mean PI lower than 3% in this study. In addition, we found no significant association between HR RR ratio and the agreement for the finger, in line with the work by Hengy et al. [12].

Various signal processing algorithms have been applied for computing PPV and PAV. To prevent displaying a spuriously high PPV value, which can, for example, be caused by an irregular beat, we adopted a 5-point median filter to smooth the PPV values. Intensive PPV post-processing can further improve the results at the cost of a clinically-relevant latency of up to 2 min, as shown by Addison et al. [20, 29]. Future research to design algorithms incurring less delay could further aid the clinical utility of PPG-derived PAV as a non-invasive measure of PPV.

The original agreement between finger-derived PAV and ABP-derived PPV found in this work (3.2 ± 5.1%) was better than that reported by Hengy et al. [13] (5.2 ± 8.4%). This may be attributed to be the fact that PPV values changed marginally for some patients in our dataset. After the baseline correction, the agreement in our study was further improved (1.2 ± 3.8%). The forehead-derived PAV initially possessed an agreement of 12.0 ± 9.1%, and was later improved to 3.3 ± 4.8% after baseline correction. This closeness in performance was also found in the trending ability before and after the baseline correction. The comparable performance at different sites is consistent with the work by Desgranges et al. [20], where the measurement was performed before and after volume expansion in well-controlled situations prior to surgery.

In addition to baseline variations, several reasons might also explain the performance difference between finger- and forehead-derived PAV in terms of approaching the value and trend of PPV. First, the difference in linearization of the received waveform between finger- and forehead-derived PPG according to Beer–Lambert law could confound the problem. Second, the transmissive and reflective modes can be associated with distinctive levels of noise, which also potentially affects their performance. Yet, the precise implementations of algorithms in commercial patient monitors are usually not known, which might account for performance difference between commercial monitors.

There are several limitations in our study. First, we did not use airway pressure or capnography signals to determine the precise timing of each ventilation cycle. Instead, we acquired the length of each cycle from the ventilator. In line with the work by Kim and Pinsky [30], we believe PPV values can be properly calculated as long as the length of the ventilation cycle is precisely determined, because the ABP and PPG signals are assumed to be stationary (the frequency components remain unchanged) in such a short time frame. Second, the method we propose to compute BV is vulnerable to noise. However, since we only used the BV values averaged on a patient basis, the potential noise problem was effectively alleviated. Furthermore, the underlying physiological mechanisms leading to difference in BV and PPV–PAV agreement have to be understood in depth, to aid the clinical application of PPG sensors for PAV monitoring. Finally, we only provided an example to illustrate the behavior of PAV in comparison to PPV in the episodes with fast hemodynamic changes, as this work focuses on overall effects on all episodes. It is of clinical interest for future research to analyze in details the behavior of PAV in different scenarios.

In conclusion, the finger-derived PAV was in better agreement with PPV, compared to forehead-derived PAV. Baseline variation was identified to be a factor significantly affecting the agreement between ABP-derived PPV and PAV calculated from finger- and forehead-derived PPG. After correcting for BV, the agreements between PPV and PAV at finger and forehead were both improved, the difference between these two agreements was diminished. The tracking abilities for both finger- and forehead-derived PAV warrant improvements for wide use in clinical practice. Overall, our results show that baseline-corrected finger- and forehead-derived PAV may provide a non-invasive alternative for PPV.
